# Antiviral Potential of Fucoxanthin, an Edible Carotenoid Purified from *Sargassum siliquastrum*, against Zika Virus

**DOI:** 10.3390/md22060247

**Published:** 2024-05-28

**Authors:** Nalae Kang, Eun-A Kim, Areumi Park, Seong-Yeong Heo, Jun-Ho Heo, Soo-Jin Heo

**Affiliations:** 1 Jeju Bio Research Center, Korea Institute of Ocean Science and Technology (KIOST), Jeju 63349, Republic of Korea; nalae1207@kiost.ac.kr (N.K.); euna0718@kiost.ac.kr (E.-A.K.); areumi1001@kiost.ac.kr (A.P.); syheo@kiost.ac.kr (S.-Y.H.); unknown0713@kiost.ac.kr (J.-H.H.); 2 Department of Biology, University of Science and Technology (UST), Daejeon 34113, Republic of Korea

**Keywords:** fucoxanthin, *Sargassum siliquastrum*, antiviral potential, zika virus

## Abstract

Considering the lack of antiviral drugs worldwide, we investigated the antiviral potential of fucoxanthin, an edible carotenoid purified from *Sargassum siliquastrum*, against zika virus (ZIKV) infection. The antiviral activity of fucoxanthin was assessed in ZIKV-infected Vero E6 cells, and the relevant structural characteristics were confirmed using molecular docking and molecular dynamics (MD) simulation. Fucoxanthin decreased the infectious viral particles and nonstructural protein (NS)1 mRNA expression levels at concentrations of 12.5, 25, and 50 µM in ZIKV-infected cells. Fucoxanthin also decreased the increased mRNA levels of interferon-induced proteins with tetratricopeptide repeat 1 and 2 in ZIKV-infected cells. Molecular docking simulations revealed that fucoxanthin binds to three main ZIKV proteins, including the envelope protein, NS3, and RNA-dependent RNA polymerase (RdRp), with binding energies of −151.449, −303.478, and −290.919 kcal/mol, respectively. The complex of fucoxanthin with RdRp was more stable than RdRp protein alone based on MD simulation. Further, fucoxanthin bonded to the three proteins via repeated formation and disappearance of hydrogen bonds. Overall, fucoxanthin exerts antiviral potential against ZIKV by affecting its three main proteins in a concentration-dependent manner. Thus, fucoxanthin isolated from *S. siliquastrum* is a potential candidate for treating zika virus infections.

## 1. Introduction

Zika virus (ZIKV), belonging to the genus Flavivirus, family Flaviviridae, is a single positive-stranded RNA virus first isolated in Uganda in 1947 which spreads via mosquitoes such as *Aedes aegypti* and *Aedes albopictus* [1,2]. Since its first large outbreak on Yap Island, Micronesia, in the Pacific Ocean, several outbreaks have occurred worldwide, including the French Polynesian Islands (2013–2014), New Caledonia (2014–2015), Brazil (2015), and several Latin American countries (2015–2016) [3,4]. In humans, ZIKV infection causes low-grade fever, rash, headache, conjunctivitis, muscle pain, and arthralgia [5]. Recently, ZIKV infection was shown to be associated with an increased incidence of congenital malformations, including microcephaly in fetuses and infants, neurological complications, and Guillain–Barré syndrome in adults [6].

The lack of diversity among antiviral drugs and agents is a major public health issue worldwide [7]. Presently, antiviral drugs clinically approved by the US Food and Drug Administration (FDA) are available for only a few viruses including human immunodeficiency virus (HIV), Hepatitis C virus (HCV), Hepatitis B virus (HBV), Influenza virus, Herpes simplex virus (HSV), Human papillomavirus (HPV), Respiratory syncytial virus (RSV), Human cytomegalovirus (HCMV), and Varicella-zoster virus (VZV); a vaccine or a clinically approved drug to treat ZIKV-infectious disease remains to be developed [8,9].

Marine organisms have evolved to produce various secondary metabolites with unique and novel components that can adapt to marine environments [10,11]. Among these, fucoxanthin is a marine carotenoid abundant in brown algae such as *Sargassum* sp. [12,13,14]. Fucoxanthin has a unique molecular structure that may exert free radical-scavenging activity, including an allenic bond, a polyene chain, an epoxy ketone group, and oxygenic functional groups such as hydroxyl and acetyl groups [15,16,17]. Fucoxanthin exhibits various functional activities, including antioxidant, anti-obesity, antidiabetic, anti-inflammatory, anticancer, and hepatoprotective activities [18]. Recently, fucoxanthin from *S. siliquastrum* was reported to exert antiviral activity against SARS-CoV-2 [12]. Fucoxanthin is also acknowledged as a dietary ingredient in a New Dietary Ingredient notification by the FDA; thus, fucoxanthin is a new potential drug candidate [17].

Therefore, this study investigated the antiviral effect of fucoxanthin, an edible carotenoid, in ZIKV-infected cells and the relevant structural characteristics using molecular docking and molecular dynamics simulation.

## 2. Results and Discussion

### 2.1. Antiviral Activity of Fucoxanthin in Zikv-Infected Vero E6 Cells

**Figure 1:**
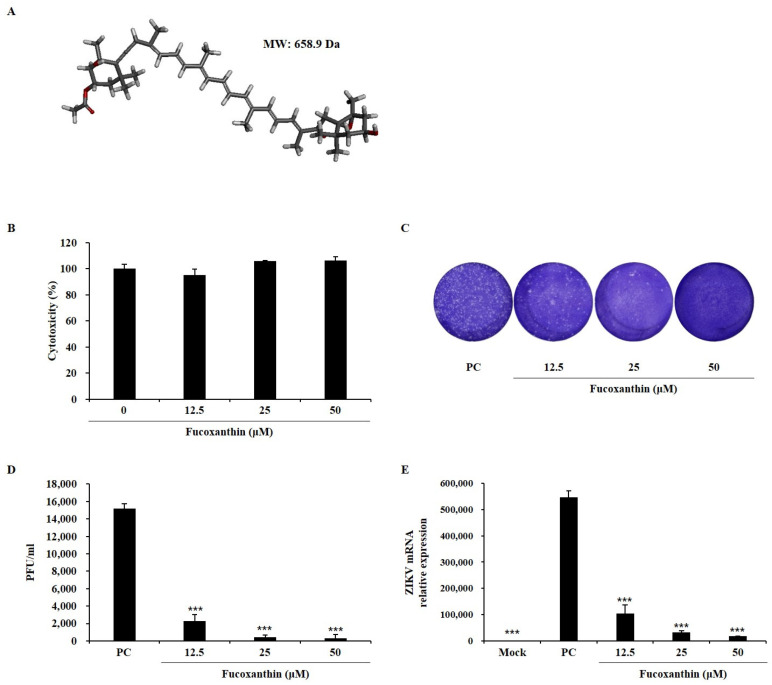
Antiviral activity of fucoxanthin in zika virus-infected Vero E6 cells. The structure of fucoxanthin (**A**), and the cytotoxicity of fucoxanthin in Vero E6 (**B**). Plaque image (**C**) and titer (**D**) on antiviral activity of fucoxanthin using plaque assay. Effect of fucoxanthin on zika virus NS1 mRNA expression level (**E**). The results are represented as the mean ± standard deviation of three determinations. *** *p* < 0.001 versus PC.

The antiviral activity of fucoxanthin was assessed based on reduced rate of viral plaque formation and nonstructural protein (NS)1 mRNA expression (Figure 1). Fucoxanthin at concentrations of 12.5, 25, and 50 µM showed no cytotoxicity (Figure 1A,B) and decreased the infectious viral particles in the media at all concentrations (Figure 1C). Infection with 0.01 MOI of virus showed a plaque-forming virus titer of 15,000 ± 566 PFU/mL, while the treatment of fucoxanthin decreased this to 2300 ± 707, 400 ± 283, and 300 ± 424 PFU/mL (Figure 1D) at 12.5, 25, and 50 µM, respectively. Further, infection with 0.01 MOI of virus increased the NS1 mRNA expression level to 500,000 times compared with that in the non-treatment (mock group), whereas fucoxanthin treatment decreased the levels to 104,168 ± 4384, 33,433 ± 1013, and 17,680 ± 1613, respectively (Figure 1E). According to the previous studies, some natural products possess the antiviral activity in ZIKV-infected cells. Berberine and emodin, isolated from herbs, show a 75–85% reduction effects at concentrations of 160 µM and 40 µM, respectively [19]. In addition, delphinidin and epigallocatechin gallate, which are types of polyphenols, exhibit virucidal effects of approximately 40% and 60%, respectively, at a concentration of 10 µM [20]. Also, pinocembrin, a flavanone, exhibits the ZIKV infection inhibition effect, showing an IC_50_ value of 17.4 µM [21]. Moreover, umifenovir, an approved drug, inhibits ZIKV, with IC_50_ values of 11 µM and 15 µM, respectively, depending on the drug treatment time (overnight and 1 h) [22]. In this study, fucoxanthin showed antiviral activity of approximately 84% at 12.5 µM, and this result indicates that fucoxanthin has a similar or high potential compared with other natural products and even an approved drug previously reported.

### 2.2. Effects of Fucoxanthin on Ifn-Induced Protein with Tetratricopeptide Repeats (Ifit) Family Expression in Zikv-Infected Vero E6 Cells

**Figure 2:**
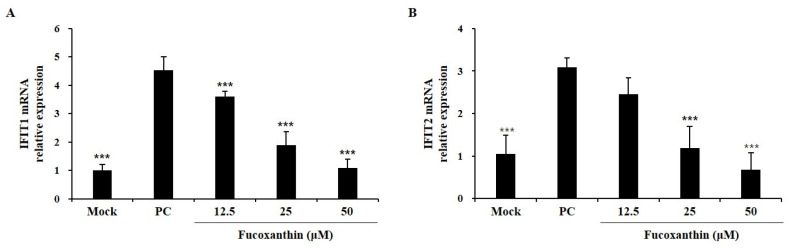
Effects of fucoxanthin on Interferons-induced protein with tetratricopeptide repeats (IFIT)1 (**A**) and IFIT2 (**B**) mRNA levels in zika virus-infected Vero E6 cells. The results are represented as the mean ± standard deviation of three determinations. *** *p* < 0.001 versus PC.

The IFIT family is well-known to be expressed in virus-infected cells and acts as an important factor in the antiviral immune response by inhibiting the translation and replication of viral proteins [23]. The effect of fucoxanthin on IFIT family members was assessed by measuring IFIT1 and IFIT2 mRNA expression levels. Infection with 0.01 MOI virus increased IFIT1 and IFIT2 mRNA levels compared to those in the non-treated group (mock group). In contrast, fucoxanthin treatment decreased these levels in a concentration-dependent manner (Figure 2). These results indicated that fucoxanthin possesses antiviral activity against ZIKV.

### 2.3. Molecular Docking Analysis of Fucoxanthin on Three Main Proteins of Zikv

To analyze the structural characteristics underlying the antiviral activity of fucoxanthin, a molecular docking analysis of fucoxanthin bound to the three main proteins of ZIKV was performed. The stabilities of the protein–fucoxanthin complexes were compared using two types of interaction energies: Chemistry at Harvard Macromolecular Mechanics (CHARMM)-based DOCKER (CDOCKER) interaction energies and calculated binding energies (Table 1). A higher -CDOCKER interaction energy and a more negative calculated binding energy suggested more favorable stability of the protein–ligand complex [24,25,26]. Fucoxanthin interacted with three main proteins: the envelope protein, NS3, and RNA-dependent RNA polymerase (RdRp), showing -CDOCKER interaction energies of 27.9351, 49.2941, and 48.5594 kcal/mol and binding energies of −151.449, −303.478, and −290.919 kcal/mol, respectively. These energy values indicated that fucoxanthin was more strongly bonded to NS3 and RdRp than to the envelope protein.

**Figure 3:**
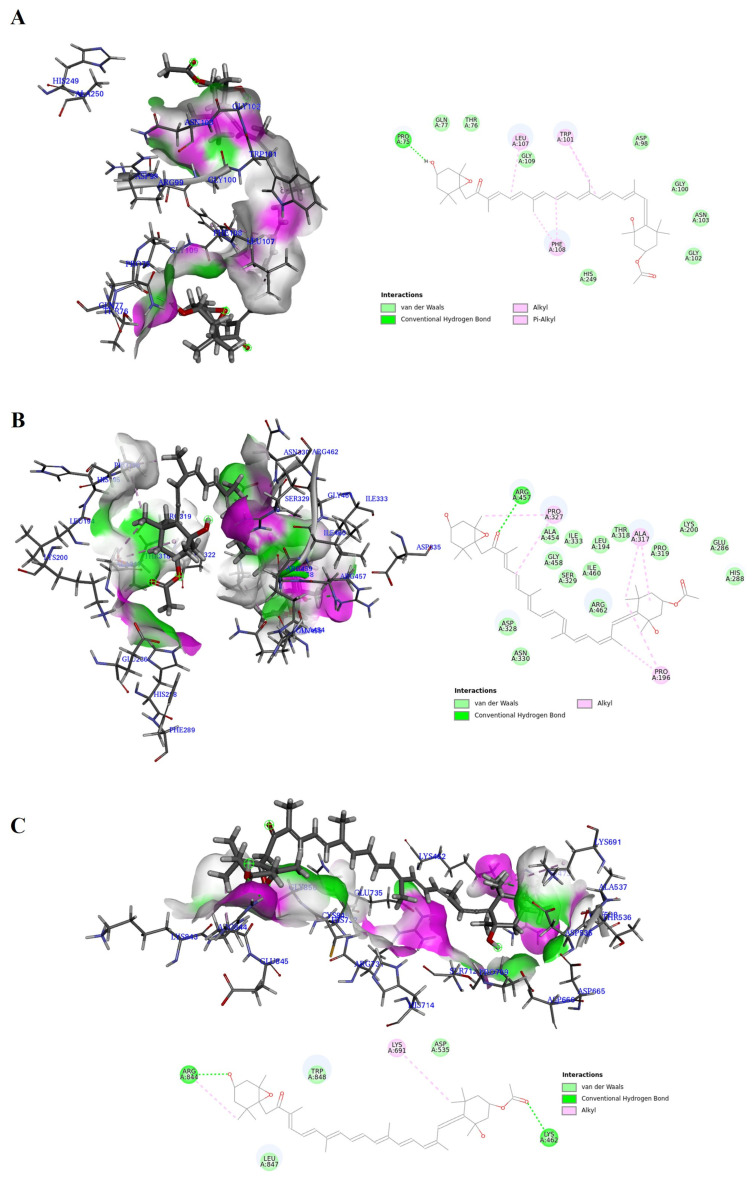
The docking poses of fucoxanthin to three main proteins of zika virus including the envelop protein (**A**), Nonstructural protein 3 (**B**), and RNA-dependent RNA polymerase (**C**).

Fucoxanthin docked in close proximity to the active site of each protein and displayed favorable bond interactions. As shown in the 3D and 2D interaction diagrams of the envelope protein–fucoxanthin complex, fucoxanthin interacted with various amino acids, including Pro75 (hydrogen bond), Trp101 (Pi-alkyl bond), Leu107 (alkyl bond), and Phe108 (Pi-alkyl bond) (Figure 3A). Fucoxanthin also formed one hydrogen and six alkyl bonds with various amino acids in NS3, including Pro196 (alkyl bond), Ala317 (alkyl bond), Pro327 (alkyl bond), and Arg457 (hydrogen bond) (Figure 3B). Furthermore, fucoxanthin interacted with amino acids of RdRp, including Lys462 (hydrogen bond), Lys691 (alkyl bond), and Arg844 (both hydrogen and alkyl bonds) (Figure 3C). These results showed that the oxygen and carbon atoms located at the end of the fucoxanthin structure played an important role in the interaction with ZIKV proteins, indicating that all the functional groups of fucoxanthin, including hydroxyl, acetyl, and epoxy ketone groups, affected its interaction with these proteins.

### 2.4. Comparison of The Zikv Protein–Fucoxanthin Complex and Zikv Protein Alone in Molecular Dynamics (Md) Simulation

**Figure 4:**
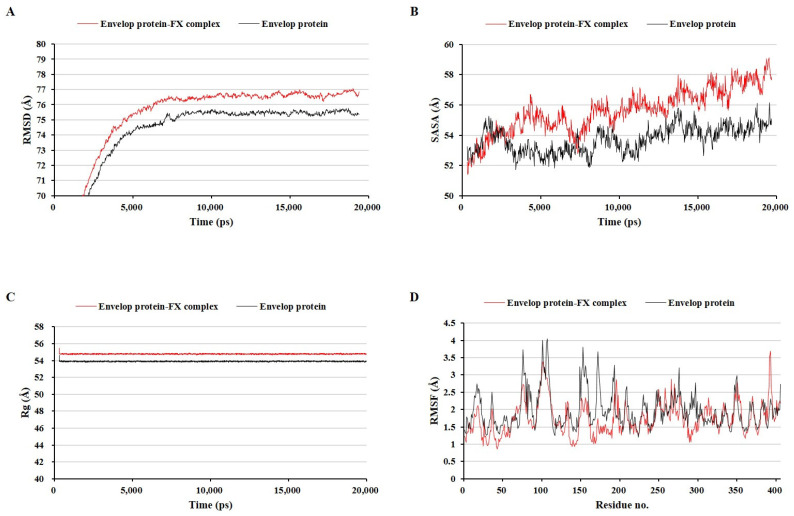
Comparison of the envelop protein–fucoxanthin complex and the envelop protein alone during molecular dynamics simulation. The root mean square deviation of the whole complex structure with water molecules (**A**), the solvent accessible surface area (**B**), the radius of gyration (**C**), and the root mean square fluctuation of amino acid residues (**D**).

**Figure 5:**
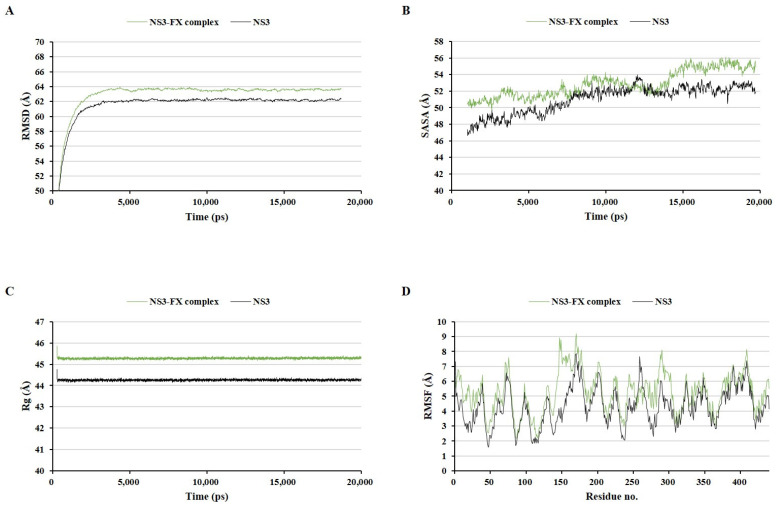
Comparison of the nonstructural protein 3–fucoxanthin complex and nonstructural protein 3 alone during molecular dynamics simulation. The root mean square deviation of the whole complex structure with water molecules (**A**), the solvent accessible surface area (**B**), the radius of gyration (**C**), and the root mean square fluctuation of amino acid residues (**D**).

**Figure 6:**
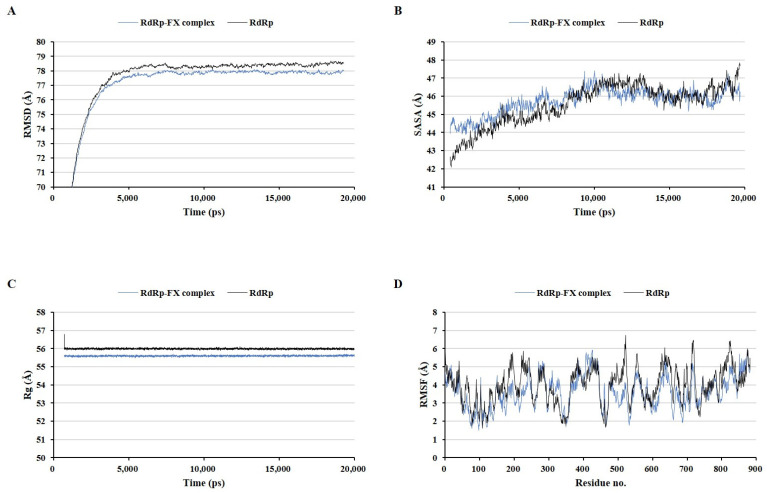
Comparison of the RNA-dependent RNA polymerase–fucoxanthin complex and RNA-dependent RNA polymerase alone during molecular dynamics simulation. The root mean square deviation on whole complex structure with water molecules (**A**), the solvent accessible surface area (**B**), the radius of gyration (**C**), and the root mean square fluctuation of amino acid residues (**D**).

MD simulations have been used in many previous studies to evaluate the stability of ligand–protein complexes [27,28]. Three protein–fucoxanthin complexes were compared to the fucoxanthin-free ZIKV protein structures under the same MD simulation conditions using four types of analyses: the root mean square deviation (RMSD) of the whole complex structure with water molecules, the solvent-accessible surface area (SASA), the radius of gyration (Rg), and the root mean square fluctuation (RMSF) of amino acid residues (Figure 4, Figure 5 and Figure 6). These factors have been used as parameters to assess the structure quality in several previous studies [29,30,31]. The envelope protein–fucoxanthin complex and the NS3–fucoxanthin complex showed similar patterns in all analyses, whereas the RdRp–fucoxanthin complex showed the opposite pattern with two protein complexes in all analyses.

The envelope protein–fucoxanthin complex showed higher RMSD, SASA, and Rg values than those of the envelope protein alone in the entire simulation period after 3000 ps (Figure 4A–C). Further, the envelope protein–fucoxanthin complex showed a lower RMSF on the active site amino acid residues, ranging from Asp98 to Gly109, than that of the envelope protein alone (Figure 4D). These results indicated that fucoxanthin stably binds to the envelope protein while maintaining the complex. The NS3–fucoxanthin complex also showed higher RMSD, SASA, and Rg values than those of NS3 alone during most of the simulation period, except at 12,000 ps (Figure 5A–C), and a generally higher RMSF on most amino acid residues of each protein than that of NS3 alone (Figure 5D). These results indicated that the NS3–fucoxanthin complex is less stable than the pure protein.

In contrast, the RdRp–fucoxanthin complex retained a lower RMSD pattern than that of RdRp alone (Figure 6A). This result indicated that the binding of fucoxanthin to RdRp increased the condensability of the protein structure. Further, the RdRp–fucoxanthin complex showed a higher SASA until 10,000 ps, but thereafter retained a similar SASA value until the end of the simulation compared to that of RdRp alone (Figure 6B). In addition, the RdRp–fucoxanthin complex maintained a lower Rg than that of RdRp alone (Figure 6C). Moreover, the RdRp–fucoxanthin complex showed a lower RMSF for most amino acid residues of RdRp, especially His714, Cys730, and Cys849, which are amino acids in the active site, than that of RdRp alone (Figure 6D). These results indicated that fucoxanthin forms a more stable complex with RdRp than with the envelop protein and NS3.

### 2.5. Md Simulation of Protein–Fucoxanthin Complexes

**Figure 7:**
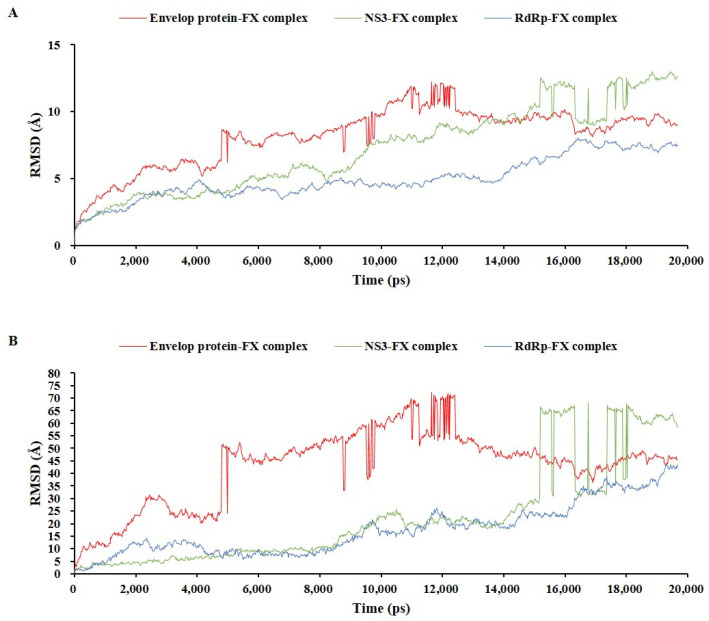
The root mean square deviation comparison of three protein–fucoxanthin complexes during molecular dynamic simulation; on the whole complex structure without water molecules (**A**) and on the interaction site between protein and fucoxanthin (**B**).

**Figure 8:**
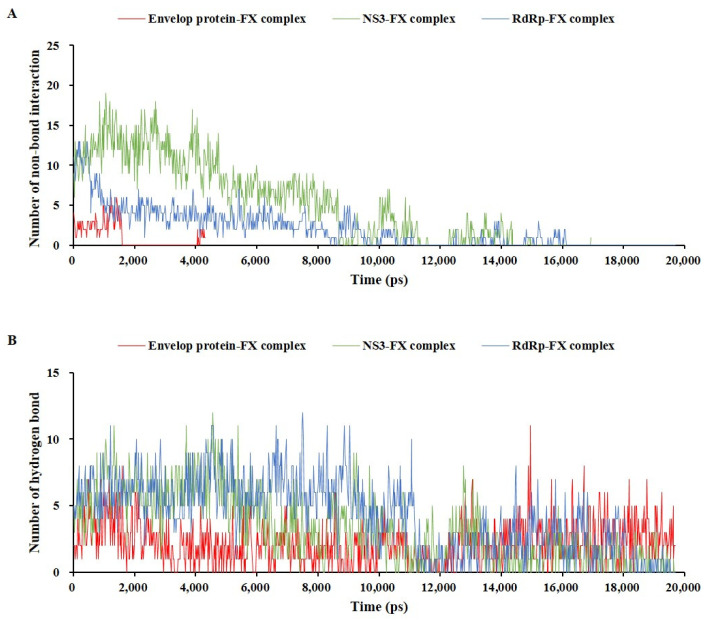
The interaction bond comparison of three protein–fucoxanthin complexes during molecular dynamic simulation; non-bond interaction (**A**) and hydrogen bond (**B**).

The stabilities of three protein–fucoxanthin complexes were analyzed and compared by simulating the biological network dynamics for 20 ns (Figure 7 and Figure 8). RMSD analysis was performed using two methods: on the whole complex structure without water molecules (Figure 7A) and on the interaction site between the protein and fucoxanthin (which indicates the ligand RMSD) (Figure 7B). In the trajectory of the whole complex structure without water molecules, the RdRp–fucoxanthin complex showed a stable RMSD in the initial frame until 14,000 ps, after which the RMSD increased. In contrast, the envelope protein–fucoxanthin complex and the NS3–fucoxanthin complex showed unstably increased RMSD values compared to that of the RdRp–fucoxanthin complex. The envelope protein–fucoxanthin complex showed a repetitive rapid change at 4900 ps, 8800 ps, 9500 ps, and a range of 11,000–12,100 ps. Further, the NS3–fucoxanthin complex showed a rapid change over a period of 15,000 ps. These rapid changes in RMSD indicated that interaction between the proteins and fucoxanthin changed rapidly (Figure 7A). In addition, as shown in Figure 7B, RMSD values of the interaction site between the protein and fucoxanthin in the three protein–fucoxanthin complexes showed similar RMSD patterns to the whole complex structure without water molecules. However, the RMSD value of the NS3–fucoxanthin complex was similar to that of the RdRp–fucoxanthin until 14,000 ps (Figure 7B), indicating that the NS3–fucoxanthin complex was influenced by water molecules to form a more stable complex. Therefore, these RMSD analysis results indicated that fucoxanthin formed a stable conformation with RdRp and NS3 rather than with the envelop protein.

The stabilities of the three protein–fucoxanthin complexes were analyzed by comparing two types of interaction bonds (Figure 8): non-bonding interactions of the complex structures without water molecules (Figure 8A) and hydrogen bonds of the complex structures with water molecules (Figure 8B). The RdRp–fucoxanthin complex and NS3–fucoxanthin complex formed and maintained non-bond interactions such as hydrogen bonding and electrostatic and hydrophobic interactions until 8000 ps, after which a few interactions repeatedly formed and disappeared, and finally completely disappeared. In particular, the NS3–fucoxanthin complex formed a larger number of non-bond interactions than those in the RdRp–fucoxanthin complex; this number of non-bond interactions in the NS3–fucoxanthin resulted in a lower binding energy for NS3–fucoxanthin (−303.478 kcal/mol). Meanwhile, the envelope protein–fucoxanthin complex showed non-bond interaction only until 1900 ps, and then formed a small number of non-bond interactions again at 4000 ps. These results indicated that the envelope protein–fucoxanthin complex maintained a weaker bond compared to that of the RdRp–fucoxanthin and NS3–fucoxanthin complexes, but had potential depending on the concentration of fucoxanthin (Figure 8A). Furthermore, the three protein–fucoxanthin complexes maintained hydrogen bonds during a simulation of 20,000 ps. The RdRp–fucoxanthin complex and NS3–fucoxanthin complex formed more hydrogen bonds than those of the envelope protein–fucoxanthin complex until 10,000 ps; in particular, all complexes maintained hydrogen bonding until 20,000 ps via repeated formation and disappearance (Figure 8B). This result indicated that fucoxanthin could affect the main proteins of ZIKV, including the envelope protein, in a concentration-dependent manner.

## 3. Materials and Methods

### 3.1. Acquisition of Fucoxanthin from S. siliquastrum

Fucoxanthin was obtained from the brown seaweed *S. siliquastrum* as previously described [32]. *S. siliquastrum* collected from Jeju Island coast, South Korea, was extracted using 80% MeOH, and the extract was partitioned with CHCl_3_. Fucoxanthin was then purified by silica open column chromatography, Sephadex LH-20 open column chromatography, and a reversed-phase high-performance liquid chromatography system (Alliance 2690, Waters corporation, Framingham, MT, USA).

### 3.2. Chemicals and Reagents

DPBS, DMEM, EMEM/F12 powder, 100× L-glutamine, and FBS were purchased from Gibco (Carlsbad, CA, USA), and Penicillin/Streptomycin and Crystal violet (C0775) were purchased from Sigma-Aldrich (St. Louis, MO, USA). TRIzol and DEPC-water were purchased from Invitrogen (Waltham, MT, USA), and chloroform, isopropanol, and ethanol were purchased from Merck & Co. (Rahway, NJ, USA). The High-Capacity RNA-to-cDNA kit and Power SYBR Green PCR Master Mix were purchased from Applied Biosystems (Waltham, MT, USA).

### 3.3. Virus and Cell Line

ZIKV was provided by the Korea Centers for Disease Control and Prevention (KCDC). Virus stock (1 × 10^5^ virus/mL) was titrated using plaque assay and stored at −80 °C. Vero E6 cells were purchased from American Type Culture Collection (Washington, DC, NW, USA). Vero E6 cells were cultured in DMEM supplemented with 10% fetal bovine serum and 1% penicillin-streptomycin, and maintained at 37 °C in a 5% CO_2_ incubator.

### 3.4. Sample Treatment and Virus Infection

Vero E6 cells were seeded in the 6-well plate at a density of 1 × 10^6^ cells/well and incubated in DMEM supplemented with 10% FBS until the cells formed a monolayer (100% confluency). The cells were washed with 1 mL of PBS, and 2 mL of DMEM supplemented with 2% FBS was added to the cells. To investigate the antiviral activity of the test sample against ZIKV, cells were treated with the test sample at concentrations of 12.5, 25, and 50 µM, and the cells were incubated for 2 h. The cells were then infected with 0.01 MOI of ZIKV. DPBS-treated cells were used as the mock group (only DPBS-treated cells) and as the positive control group (virus-infected cells). Sample/DPBS-treated cells were incubated for 48 h. The sample/DPBS-treated cell media (supernatant) were used for the plaque assay to titrate the number of virus particles, and the sample/DPBS-treated cells were used for the qPCR assay to assess the mRNA levels related to ZIKV.

### 3.5. Plaque Assay

Each sample/DPBS-treated cell medium was serially diluted 10-fold in a 1.5 mL tube with DMEM alone. Vero E6 cells were seeded in the 6-well plate at a density of 1 × 10^6^ cells per well and incubated in DMEM supplemented with 10% FBS until the cells formed a monolayer (100% confluency). The cells were washed twice with 1 mL of DPBS, and 0.2 mL of serum free-DMEM and each sample/DPBS-treated cell media dilution (0.5 mL) was added to the respective cells to the wall of the well. The cells were incubated at 37 °C in a 5% CO_2_ incubator for 2 h with gentle shaking every 15 min to allow virus adsorption. After adsorption, the inoculum was removed from the cells, and 3 mL of pre-mixed DMEM-F12-2% oxoid agarose (at a ratio of 7:3) was added to the cells and incubated at 37 °C in a 5% CO_2_ incubator for 5 days. The cells were fixed with 1 mL of 4% formaldehyde for 1 h. After removing the 4% formaldehyde, the agarose gel was removed, and the cells were stained with 1 mL of 0.1% crystal violet for 30 min at room temperature. The crystal violet solution was then discarded and the cells were washed with DPBS and dried. Virus titers were calculated by counting the number of plaques using the following formula:

PFU/mL=Number of plaques/(dilution factor×volume of diluted virus/well)

### 3.6. q-PCR

RNA was extracted using a guanidinium thiocyanate-phenol-chloroform extraction method. TRIzol (1 mL) was added to the sample/DPBS-treated cells, and the mixed TRIzol was harvested in a 1.5 mL e-tube. Then, 200 µL of chloroform was added, and the e-tube was vortexed for 15 s. After incubation for 3 min at RT, the tube was centrifuged with 12,000 rpm for 15 min at 4 °C. Then, 500 µL of the aqueous layer was transferred to a new e-tube, and 500 µL of iso-propanol was added and inverted 5–6 times. After incubation for 10 min at RT, the tube was centrifuged with 12,000 rpm for 10 min at 4 °C. The pellet was then washed using 1 mL of EtOH and centrifugation (12,000 rpm for 10 min at 4 °C). Finally, the RNA pellet was dried, dissolved using 25 µL of DEPC-water, and stored at −80 °C. cDNA synthesis was performed using a High-Capacity RNA-to-cDNA kit following the manufacturer’s instructions. A total of 2 µg of RNA was mixed with 2× RT buffer mix and 20× RT enzyme mix, incubated for 60 min at 37 °C, and then inactivated for 5 min at 95 °C. cDNA was stored at −80 °C. q-PCR was performed using the SYBR green-based detection method. cDNA was diluted to 1/40 and mixed with SYBR Green and the target primers following the appropriate ratio in a 96-well real-time PCR plate. All primers used in this study were obtained from a previous study [33] and are listed in Table 2.

### 3.7. Statistical Analysis

All data were represented as the mean ± standard deviation of three determinations. Statistical comparisons were performed using one-way ANOVA, followed by Dunnett’s multiple comparison test, using GraphPad Prism software version 9 (GraphPad Software, San Diego, CA, USA). Statistical significance was set at *p* values < 0.05 and 0.001.

### 3.8. D Structure of Fucoxanthin

The structural data file of fucoxanthin was obtained from PubChem (CID 5281239), and geometry optimization of the fucoxanthin 3D structure was performed using the ligand preparation, energy minimization, and conformation generation protocols of Discovery Studio 2023.

### 3.9. D Structure of the Major ZIKV Proteins

For molecular docking studies, 3D structures of the main ZIKV proteins were obtained from Protein Data Bank (PDB). The envelope protein (PDB ID: 5JHM), NS3 (PDB ID: 5GJB), and RdRp (PDB ID: 5TFR) structures had a resolution of 2.00 Å, 1.70 Å, and 3.05 Å, respectively. The 3D structures were prepared using the protein preparation protocols and optimized using MD simulation protocols in Discovery Studio 2023. The binding site of the envelope protein was defined from the fusion loop, the binding site of NS3 was defined from the site interacting with adenosine triphosphate, and the binding site of RdRp was defined from the priming loop based on the previous papers [34,35,36,37].

### 3.10. Molecular Docking Analysis between the Protein and Fucoxanthin

Molecular docking analysis was performed to assess the structural effects of fucoxanthin on the three main proteins using the CDOCKER protocol based on the CHARMM and Calculate Binding Energies tools in Discovery Studio 2023 (Biovia, San Diego, CA, USA). The sphere of the active site was extended to a radius of 15.5 Å considering the distance from fucoxanthin. The docking pose of fucoxanthin on the three main proteins was expressed as 2D diagrams and 3D crystal structures.

### 3.11. MD Simulation

MD simulations were performed using the CHARMM force field in Discovery Studio 2023 to investigate the dynamic behavior of each complex of the three main proteins and fucoxanthin. Each step of the MD process was conducted according to the following simulation protocols [25,38,39]: solvation with an explicit periodic boundary; 1000 steps of minimization using the steepest descent algorithm; 2000 steps of minimization using the adopted basis Newton–Raphson algorithm; 300 ps heating at 300 K; and 19,700 ps production in a standard number of particles, pressure, and temperature ensemble. The simulation was performed with a time step of 2 fs, and the trajectory frame was collected every 2 ps.

## 4. Conclusions

Unfortunately, no drug has yet been approved for treating ZIKV infection. Therefore, exploring potential drugs for treating ZIKV infection is essential. To confirm the potential of fucoxanthin as an antiviral drug derived from natural marine organisms, its antiviral activity was evaluated both in vitro in ZIKV-infected Vero E6 cells and in silico. Fucoxanthin decreased the infectious viral particles and NS1 mRNA expression levels in ZIKV-infected Vero E6 cells. In silico molecular docking and MD simulations predicted that fucoxanthin stably bound to three main proteins of ZIKV including the envelope protein, NS3, and RdRp, depending on the fucoxanthin concentration. Therefore, fucoxanthin is a potential antiviral drug against ZIKV. However, further studies, including absorption, distribution, metabolism, excretion, and toxicity for in vivo analyses, are required to industrially utilize fucoxanthin.

## Figures and Tables

**Table 1:** Calculated binding energies of fucoxanthin on three main proteins of zika virus.

ZIKV Proteins	-CDOCKER Interaction Energy (kcal/mol)	Binding Energy (kcal/mol)
Envelope protein	27.9351	−151.449
NS3	49.2941	−303.478
RdRp	48.5594	−290.919

**Table 2:** The information of primers.

Gene	Sequence	Primer
ZIKV NS1	5′-CRA CTA CTG CAA GYG GAA GG-3′	F
	5′-GCC TTA TCT CCA TTC CAT ACC-3′	R
Monkey IFIT1	5′-GGA TTC TGT ACA ATA CAC TAG AAA CCA-3′	F
	5′-CTT TTG GTT ACT TTT CCC CTA TCC-3′	R
Monkey IFIT2	5′-ATC CCC CAT CGC TTA TCT CT-3′	F
	5′-CCACCTCAATTAATCAGGCACT-3′	R

## Data Availability

The original contributions presented in the study are included in the article, further inquiries can be directed to the corresponding author.
